# Cellular origins of mucinous ovarian carcinoma

**DOI:** 10.1002/path.6407

**Published:** 2025-03-03

**Authors:** Nicola S Meagher, Martin Köbel, Anthony N Karnezis, Aline Talhouk, Michael S Anglesio, Andrew Berchuck, Simon A Gayther, Paul PD Pharoah, Penelope M Webb, Susan J Ramus, Kylie L Gorringe

**Affiliations:** ^1^ The Daffodil Centre The University of Sydney, a joint venture with Cancer Council NSW Sydney New South Wales Australia; ^2^ School of Clinical Medicine, UNSW Medicine and Health University of NSW Sydney Sydney New South Wales Australia; ^3^ The University of Calgary Alberta Calgary Canada; ^4^ University of California Davis Sacramento CA USA; ^5^ University of British Columbia Vancouver British Columbia Canada; ^6^ Department of Obstetrics and Gynecology, Division of Gynecologic Oncology Duke University Medical Center Durham NC USA; ^7^ University of Texas Health Science Centre San Antonio San Antonio TX USA; ^8^ Department of Computational Biomedicine Cedars‐Sinai Medical Centre Los Angeles CA USA; ^9^ QIMR Berghofer Medical Research Institute Brisbane Queensland Australia; ^10^ Adult Cancer Program, Lowy Cancer Research Centre University of NSW Sydney New South Wales Australia; ^11^ The Sir Peter MacCallum Dept of Oncology University of Melbourne Melbourne Victoria Australia; ^12^ Peter MacCallum Cancer Centre Melbourne Victoria Australia

**Keywords:** ovarian cancer, cell of origin, mucinous

## Abstract

Mucinous ovarian carcinoma (MOC) is a rare histotype of epithelial ovarian cancer. Its origins are obscure: while many mucinous tumours in the ovary are metastases from the gastrointestinal tract, MOC can occur as an ovarian primary; however, the cell of origin is not well established. In this review we summarise the pathological, epidemiological, and molecular evidence for the cellular origins of MOC. We propose a model for the origins of the various tumours of the ovary with mucinous differentiation. We distinguish Müllerian from gastrointestinal‐type mucinous differentiation. A small proportion of the latter arise from teratoma and a distinct terminology has been proposed. Other gastrointestinal mucinous tumours are associated with Brenner tumours and arise from their associated benign lesions, Walthard nests. The remaining mucinous tumours develop either through mucinous metaplasia in established Müllerian tumours or with even greater plasticity through gastrointestinal metaplasia of epithelial or mesothelial ovarian inclusions. This model remains to be validated and mechanistically understood and we discuss future research directions. © 2025 The Author(s). *The Journal of Pathology* published by John Wiley & Sons Ltd on behalf of The Pathological Society of Great Britain and Ireland.

## Introduction

Mucinous ovarian carcinoma (MOC) is a rare ovarian cancer histotype with obscure cellular origins. MOCs develop in the ovary mostly in a well‐characterised progression from a benign mucinous tumour through to carcinoma via a ‘borderline’ tumour intermediate [1]. While the cellular origins of other ‘ovarian’ carcinoma histotypes appear to be in either the fallopian tube (e.g. high‐grade serous carcinomas, HGSC) or endometrial cells via endometriosis (e.g. clear cell and endometrioid carcinomas) [2]; the cellular origin of MOC remains unknown. MOC appear to be of the gastrointestinal cell type [3], but after recognition that many ‘MOC’ were in fact metastases from the colon, appendix, or pancreas [4], there was a period when many primary MOC were erroneously flagged as potential metastases. However, the genetic, anatomic, histopathologic, and outcome data from benign and borderline mucinous tumours support a nonmetastatic origin for primary MOC [5]. With improved histological and imaging tools, this issue has been somewhat resolved, yet occasionally it can be challenging to be certain of an ovarian origin.

Knowing the origins of a cancer type has important clinical and research implications. For HGSC, it has influenced early detection and prevention strategies such as opportunistic salpingectomy [6]. It also changes the treatments available, since many chemotherapies are prescribed based on the organ of origin, and many more recent targeted therapies are only approved for certain tumour types. This issue is highly relevant for MOC, given its known intrinsic resistance to standard‐of‐care ovarian cancer chemotherapy and the lack of any effective second and later‐line options [7]. Although MOC is sometimes treated based on the morphological resemblance to gastrointestinal tumours, it is unclear whether these therapies are any more effective [8, 9, 10]. Thus, knowing the true cellular lineage of MOC could open up novel avenues for risk prediction, prevention, early intervention, and/or therapy.

In this review we explore the various lines of evidence that have been used to evaluate the possible cellular origin of MOC. We draw upon published and unpublished data from genetic association studies, epidemiology, somatic genetic and gene expression profiling, histopathology analyses, and both *in vitro* and *in vivo* model systems. We also suggest avenues for future research to close the gap in our understanding of the aetiology of this disease.

## Epidemiology

### Incidence and survival

Globally, 314,000 ovarian cancers are diagnosed each year [11]. Epithelial ovarian carcinomas (EOC) comprise ~90% of these, and MOC comprises 3%–4% of all EOC [12]. This proportion was previously reported to be higher at around 11% [13, 14]. However, it is now widely accepted that many historical series contained patients with metastases to the ovary, often from the gastrointestinal tract, that were misclassified as MOC [15]. Unlike the most common histotype of EOC, HGSC, most patients with MOC are diagnosed at an early stage [16] and younger age (mean 54 years, versus 61 years for HGSC) [17, 18]. Benign or mucinous borderline tumours (MBT) are more frequent than MOC, and patients with MBT have an excellent prognosis (5‐year overall survival 97%) [19, 20]. In the Ovarian Tumour Tissue Analysis (OTTA) consortium, which included 214 MOC, the unadjusted 5‐year overall survival was 80% in cases with Stage I/II disease versus 17% in cases with Stage III/IV disease (*p* < 0.001) [21]; this is similar to reports from Surveillance, Epidemiology, and End Results (SEER) cancer registry data, comprising 2,782 MOC cases [18].

Some notable racial and ethnic differences exist in the distribution of EOC histotypes. For MOC, a higher prevalence has been observed in Native Hawaiian/Pacific Islander (13.1%) [22] and Asian patients (10.8%), compared with 8.3% in Hispanic and 4.8% of non‐Hispanic White women [23]. It is unclear whether the higher frequency of MOC in these racial and ethnic groups, which have historically been underrepresented in ovarian cancer studies, relates to genetic differences or variation in known risk factor prevalence in these populations, warranting further work.

The best‐supported model of tumourigenesis for MOC is along a continuum from benign mucinous cystadenoma, to MBT, through to mucinous adenocarcinoma. Evidence supporting this model includes shared common low penetrance risk variants, early molecular events, acquisition of additional genetic events along the continuum, the coexistence of benign, borderline, and invasive components within tumours from the same patient and shared epidemiology [24].

### Epidemiologic risk factors

Several large, pooled epidemiological studies have examined risk factors across the histotypes of EOC. Notably, some of the well‐established associations between reproductive and hormonal factors including parity, oral contraceptive use, breastfeeding, and tubal ligation and a reduced risk of ovarian cancer overall, appear to be weaker or absent for MOC, particularly when compared to endometrioid and clear‐cell carcinoma [25, 26]. Likewise, in contrast to high‐grade serous and endometrioid carcinomas, the use of menopausal oestrogen therapy has not been associated with the risk of MOC [26, 27]. A clear association with a family history of EOC has also not been observed [28].

In contrast, in both the Ovarian Cancer Association Consortium (OCAC) [29] and the Ovarian Cancer Cohort Consortium (OC3) [26], a 30%–50% increased risk of MOC was observed among current smokers, with stronger associations seen for borderline tumours. Similar associations have been reported for benign mucinous tumours, supporting an etiological continuum through to carcinoma [30]. The increase in risk with smoking is not observed for the other major histotypes. Smoking has also been associated with poorer survival after a diagnosis of ovarian cancer, particularly for MOC [31, 32]. Body mass index (BMI) is another risk factor that varies by histotype [26, 33]. Risk of MOC increases by 10%–20% per 5 kg/m^2^ increase in BMI, with significant but weaker associations (5%–10% per 5 kg/m^2^) for borderline mucinous tumours [34]. A similar trend has also been reported for benign mucinous tumours [30].

Collectively, the differences in lifestyle risk factors for MOC compared to the other EOC histotypes suggest a reduced influence of reproductive factors, which could be due to differences in the cell of origin of disease and a relatively lower reliance on hormone‐stimulated growth, or a lack of power to detect weaker effects. Indeed, oestrogen and progesterone receptor expression are generally low or absent in MOC, in contrast to the serous and endometrioid histotypes [35]. Other smoking‐related cancers include pancreatic, bladder, gastric, colorectal, and cervical cancers [36], and obesity is commonly associated with endometrial cancer [37]. Notably, most risk factors are shared between MOC and MBT, and, when this has been studied, benign mucinous ovarian tumours, suggesting that undue admixture of misdiagnosed metastases from other organs is unlikely to be a major reason underlying these findings. Due to the rarity of MOC, there may be lifestyle or environmental risk factors that cohort and case–control studies have been underpowered to detect to date.

### Genetics and the relationship to cell of origin

The prevalence of germline genetic features can also be used to infer the cell of origin of disease. Unlike the most common histotype, HGSC, there have not been any high‐ or moderate‐risk gene mutations associated with inherited predisposition to MOC [38]. Despite early reports associating MOC with Lynch syndrome [39], these have not been validated in more recent studies with appropriate pathology review [40, 41, 42, 43, 44], suggesting that earlier associations may have been related to misdiagnosed metastatic endometrial and colorectal mucinous tumours, or misclassified endometrioid ovarian carcinomas [35].

The five major histotypes of invasive EOC and the two main borderline histotypes share a substantial component of heritable risk. Nevertheless, there are differences in genetic associations, which suggest that there may be differences in the cell of origin of the different histotypes. MOC is the most genetically distinct of the histotypes—the genetic correlation between MOC and HGSC, low‐grade serous ovarian carcinoma, endometrioid ovarian carcinoma, and clear‐cell ovarian carcinoma is low (linkage disequilibrium score regression of 0.24, 0.21, 0.23, and −0.21 respectively), whereas the genetic correlations between pairs of the other histotypes is higher (ranging from 0.44 to 1) [45].

**Figure 1:**
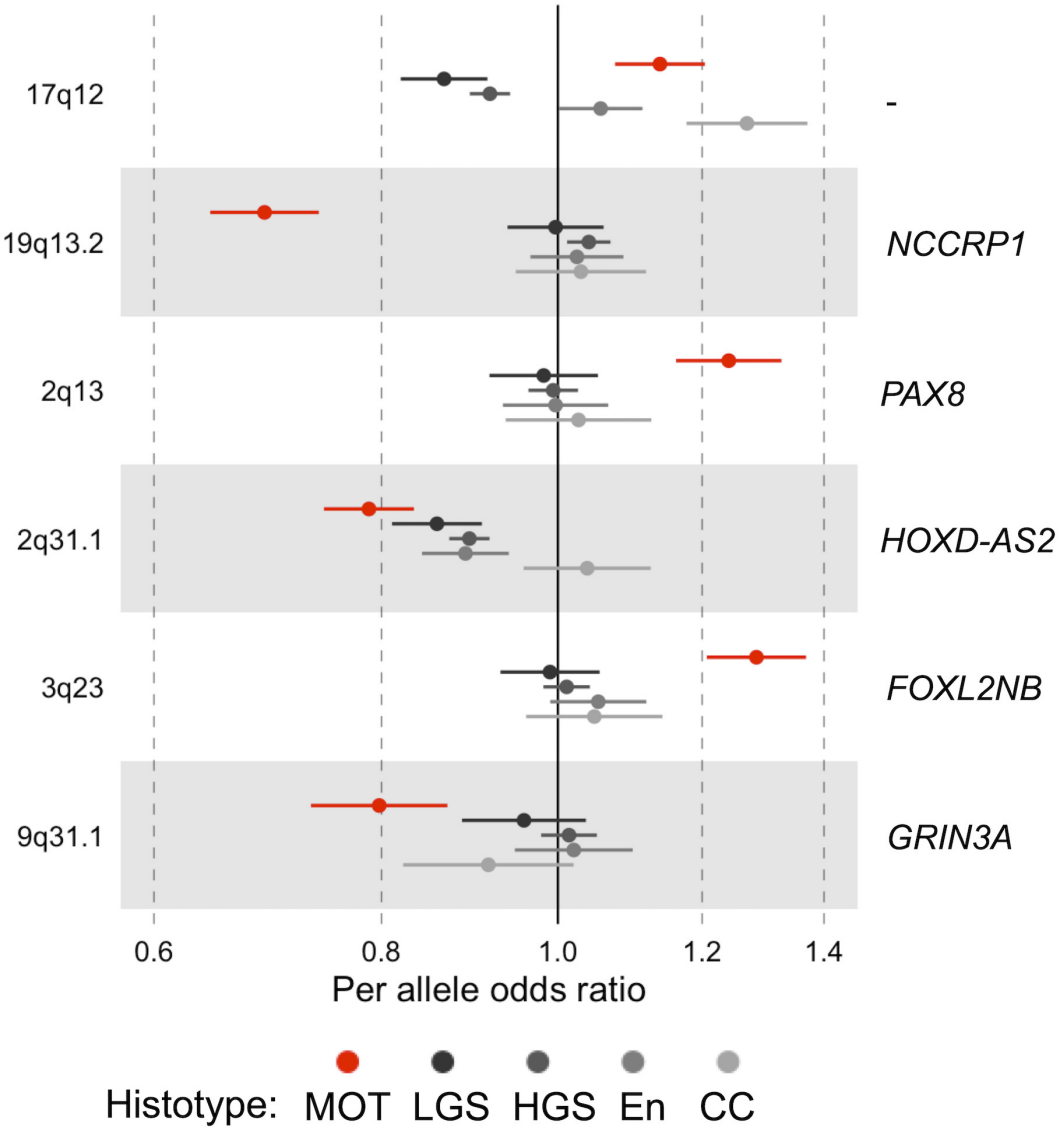
Top loci associated with the mucinous histotype identified in GWAS studies. The SNPs at loci 19q13.1, 2q13, and 3q23 are unique to mucinous ovarian tumours (MOT, including mucinous ovarian carcinoma (MOC) and mucinous borderline ovarian tumours, MBT); while others are shared to some extent with the other histotypes such as: LGS (low‐grade serous ovarian carcinoma); HGS (high‐grade serous ovarian carcinoma); En (endometrioid ovarian carcinoma); CC (clear‐cell ovarian carcinoma). Odds ratio shown is for the minor allele. The candidate genes shown are those with the highest combined score for the gene, transcriptome and chromatin analyses performed by Dareng *et al* [45]. 17q SNPs for MOC were not included in that analysis.

The most recent and largest genome‐wide association (GWAS) study reported by the international Ovarian Cancer Association Consortium reported 27 common genetic variants associated with at least one of the major histotypes of nominal genome‐wide significance [45]. In that study there were 1,149 MBT and 1,417 MOC, and the identified risk single nucleotide polymorphisms (SNPs) were associated with both tumour types (supplementary material, Table S1). This observation provides further evidence in support of the invasive cancers arising from borderline cases rather than being metastatic to the ovary. Thus, MOC and MBT were combined in other analyses, as there was little evidence for any genetic differences between them. There were six SNPs associated with the combined mucinous histotype at a significance threshold of *p* < 10^−5^, which is less stringent than the genome‐wide threshold of <5 × 10^−8^. Of these, only three were also associated with other histotypes [45] (Figure 1). Moreover, a polygenic risk model for HGSC [46] was not associated with MOC and a polygenic model for MOC was not associated with HGSC. Overall, genome‐wide SNP‐heritability was estimated to be 1.6% for MOC [45].

Transcriptome‐wide association (TWAS) analysis combined with chromatin interaction data for fallopian tube cells identified that the *HOXD* cluster on 2q31.1 was shared between MOC and the other histotypes, confirming an older study [47], although the causal variants in MOC seemed to be located in a long noncoding gene nearby (*LINC01116*) [45]. The 2q13 locus is also associated with different causal SNPs to the other histotypes by fine mapping, but remains focussed on *PAX8* as the most likely gene. The consistent genetic association with *PAX8* is intriguing, given that MOC seldom strongly express the protein. More functional work is required to better understand this connection. Similarly, deeper analysis of the MOC‐specific SNPs and associated genes may yield further insights into a cell of origin.

Shared epidemiological factors may suggest a shared mechanism or cell of origin for cancers of different types. For example, clear cell and endometrioid OC are strongly associated with endometriosis and have shared GWAS variants, linking them to their highly likely shared tissue of origin (endometrial epithelial cell types via endometriosis) [48, 49]. A recent analysis seeking a shared signal between endometrial cancer and ovarian carcinoma GWAS hits did not identify any MOC/MBT‐ascertained variants that were shared with endometrial carcinoma [50]. No similar studies have been done yet with other cancer types such as gastrointestinal tumours.

In conclusion, genetic analyses of MOC and MBT suggest that some low penetrance susceptibility alleles specific to MOC/MBT may drive disease development and can be used to indicate the cell of origin of the disease. It is particularly notable that the genome‐wide significant associations that are observed were identified in only a relatively small number of MOC compared to HGSC, and that these associations became stronger when MOC were combined with MBT, suggesting a level of homogeneity in the case series that indicates that these genetic associations are real.

### Histopathological features of mucinous tumours

Mucinous tumours are typically composed of gastrointestinal cell types showing gastric foveolar morphology, with a subset containing MUC2+ goblet cells [51]. Of note, tumours that show hormone receptor‐positive endocervical mucinous differentiation, which are often associated with endometrioid lesions, are now considered seromucinous tumours or endometrioid neoplasms with mucinous differentiation [52]. Their histomorphology resulted in frequent misclassification, attracting diagnoses including mucinous, endometrioid, and low‐grade serous. Molecular and immunohistochemical characterisation now shows these are dominantly a heterogeneous group within the spectrum of endometrioid ovarian carcinomas [35, 52]. These tumours are not the subject of this review, but it is likely that a proportion of historical MOC cases includes this misdiagnosed type [41].

In accordance with a progression model, MOC is often histologically heterogeneous, displaying areas of benign mucinous cystadenoma, borderline, and carcinoma, requiring standardised sampling for accurate diagnosis [53]. Benign mucinous cystadenomas are characterised by bland basal nuclei with abundant apical gastric foveolar mucin [3], while borderline tumours exhibit a higher degree of architectural complexity with varying mitotic activity and usually low‐grade nuclear atypia in the absence of intraepithelial carcinoma. MOC, by contrast, shows invasion of at least 5 mm [51]. There are two categories to the pattern of invasion: confluent (or expansile) and infiltrative (or destructive), the latter conferring a more aggressive disease [54, 55, 56, 57, 58]. Patients diagnosed with MOC of the infiltrative type of invasion have a higher risk of death within 2 years of their diagnosis, even if initially diagnosed at a low stage [55, 56]. Indeed, the European treatment consensus guidelines suggest that chemotherapy should be considered for any patient with Stage I MOC with an infiltrative pattern of invasion, as opposed to observation for those with an expansile pattern [59].

Recent studies have assessed the diagnostic reproducibility of MBT and MOC diagnoses as well as the patterns of invasion. The diagnostic agreement for MBT versus MOC was only moderate, with one study showing that the use of ancillary p53 immunohistochemistry (IHC) in difficult cases improved the agreement to substantial [60, 61, 62, 63]. The reproducibility of the pattern of invasion was also only moderate, and two major causes were identified [55]. First, some MOC show a so‐called adenofibromatous pattern that does not fit exactly into the confluent or infiltrative categories, but behaves more like the confluent pattern and should not be diagnosed as infiltrative [55]. Second, because the infiltrative pattern can arise in a confluent MOC, creating a combined pattern, the classification of confluent MOC with minor foci of a possible infiltrative pattern is irreproducible. A minimum of 10% or 1 mm in linear extent of infiltration has been suggested to assign an otherwise confluent MOC to the infiltrative pattern [54, 55, 57]. That the infiltrative growth pattern can arise within confluent MOC supports a shared aetiology of both patterns. It would be challenging to undertake sufficiently well‐powered epidemiological or GWAS to identify host or environmental triggers for developing the infiltrative pattern, given that this comprises 18%–25% of an already rare disease.

One of the important issues in the diagnosis of MOC, particularly in higher‐stage (III/IV) disease, can be the misclassification of primary disease versus metastases that involve the ovaries but have arisen from other sites. The most common primary mucinous tumours that metastasise to the ovary include the lower gastrointestinal tract (colon/appendix; ~45%), upper gastrointestinal tract (pancreas/stomach/biliary; 20%), and uterus (cervix/endometrium; 18%) [64]. Sometimes, the ovarian tumour is the initial presentation. In this setting, characteristics of the tumour presentation can aid diagnosis. Stage I, primary MOC is more likely to have a tumour size greater than 10 cm (mean 20 cm) [64] and show unilateral involvement (79%), whereas metastases to the ovary from elsewhere tend to be smaller (mean 7–10.6 cm), present in both ovaries, and display a nodular infiltrative growth pattern with ovarian surface involvement [64, 65]. The National Comprehensive Cancer Network (NCCN) clinical guidelines suggest routine abdominal imaging and upper and lower endoscopy to rule out a gastrointestinal primary as well as measuring CEA and CA19‐9 levels [66]. An observation that strongly favours a primary MOC is an associated teratoma, while signet ring cells (Krukenberg tumour) strongly favour a gastrointestinal (usually gastric) origin [67].

Immunohistochemical panels can also aid the diagnosis of primary compared to metastatic MOC. Markers that most efficiently distinguish ovarian from lower gastrointestinal metastases (with an accuracy of 95%) are CK7 and SATB2 [21]. Pancreatic adenocarcinomas are especially challenging, as they can mimic primary MOC when they metastasise to the ovaries and may even contain benign and borderline‐appearing areas [68]. Ancillary IHC markers such as CK17 and SMAD4 have shown some promise in distinguishing ovarian mucinous tumours from metastatic pancreatic ductal adenocarcinomas [69, 70]; however, the accuracy as individual markers remains limited, and further research into a marker panel is required. With growing pathological and clinical efforts, the misclassification rate of stage III/IV MOC has decreased significantly in recent years, enabling a more accurate study of the disease while facing the challenge of the increasing rarity of clinical trials. Furthermore, one has to keep in mind that the prognosis of stage III/IV MOC is similarly dismal compared to metastases to the ovary [15, 71].

Pseudomyxoma peritonei (PMP) is a specific condition characterised by tumour cells producing an abundance of extracellular mucin. It is most commonly associated with low‐grade appendiceal mucinous neoplasms (LAMN), which indicate metastatic disease to the ovary [72]. However, the rare exception is that a low‐grade ovarian mucinous neoplasm (LOMN) arising in a teratoma can mimic an LAMN by morphology and immunophenotype [3]. Previously, it was recommended that appendectomy be performed in all cases with a frozen section of MOC, but contemporary recommendations (NCCN) suggest removal only if it is grossly abnormal [66].

While the histopathological features of MOC have established the benign‐borderline‐carcinoma progression model, they are not able to pinpoint a cell of origin once the metastatic tumours from other organs are excluded.

## Molecular landscape

### Genomic alterations

**Figure 2:**
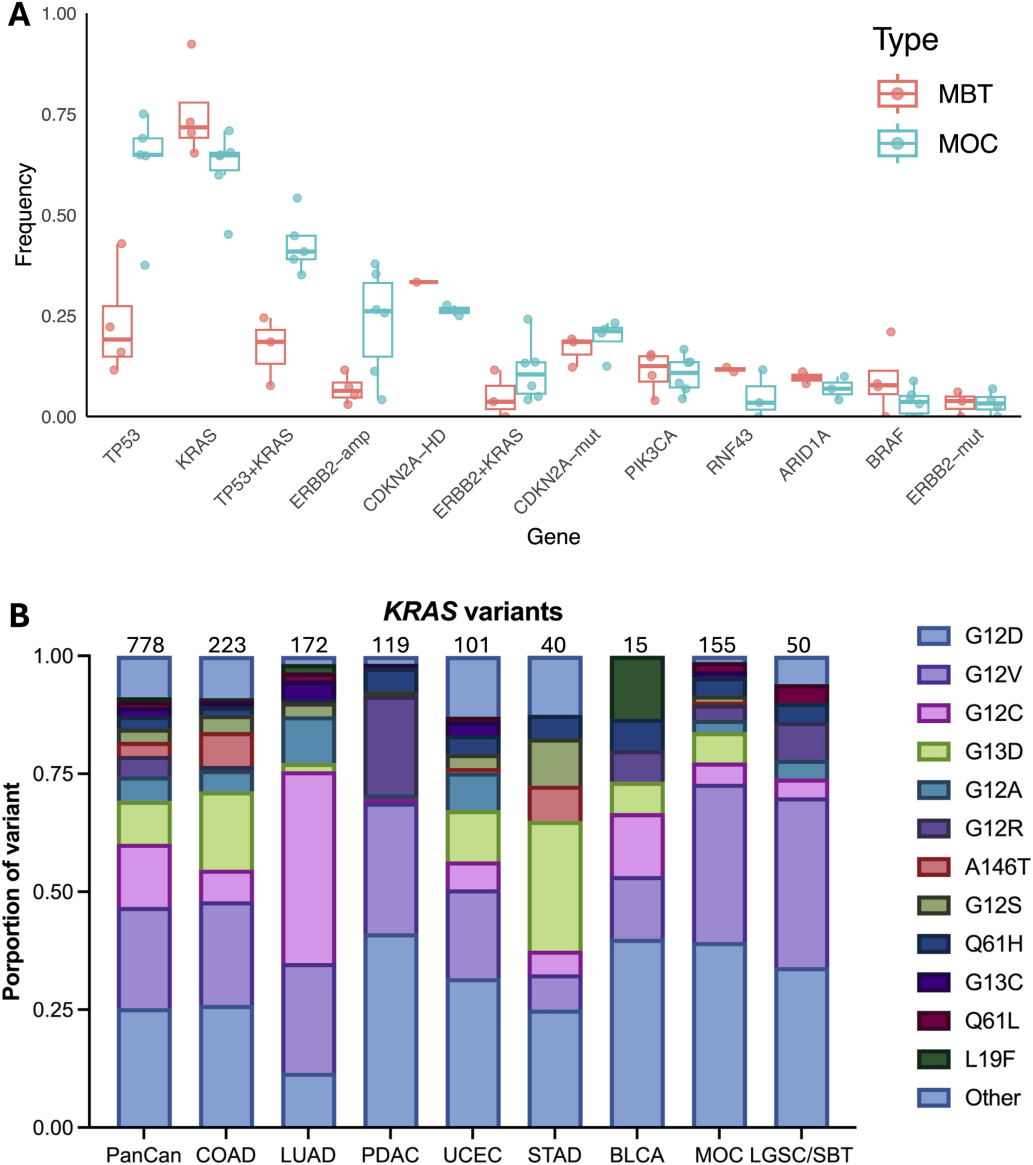
Mutations in MOC. (A) Frequency of genetic events in mucinous borderline tumours (MBT) and mucinous ovarian carcinoma (MOC). Each dot represents the frequency of the genetic event in one of the studies listed in Table 1. Not all studies had data for all genes. Amp = amplified, HD = homozygous deletion, mut = mutated; mutated only for other genes. Box and whiskers show median, quartiles and 1.5× interquartile range. (B) Frequency of *KRAS* variant types in selected cancers from TCGA Pan‐Cancer [79], mucinous ovarian carcinoma (MOC) [5] and low‐grade serous ovarian carcinoma (LGSC)/serous borderline ovarian tumours (SBT) [80, 81, 82]. Numbers of variants are given above each bar.

MOC has a distinct pattern of genomic alterations compared to the other ovarian carcinoma histotypes. *KRAS* and *TP53* mutations are almost mutually exclusive in serous carcinomas, being common in the low‐grade and high‐grade histotypes, respectively. However, co‐occurring mutations in *KRAS* and *TP53* are frequent events in MOC (35%–54% co‐occurring, Figure 2A), similar to pancreatic adenocarcinomas [83]. One study in a Singaporean population found a lower proportion of tumours with *KRAS* mutations (45%) compared to other studies (~60%) [78]. Other key alterations include *CDKN2A* (including homozygous deletions) (range 34%–50%) and amplifications in *ERBB2* (range 4%–38%, median 26%). Less common events include mutations in *RNF43* (3%–12%), *BRAF* (0%–9%), *PIK3CA* (4%–17%), and *ARID1A* (4%–10%) (Table 1, Figure 2A). Genetic events that statistically significantly co‐occur in MOC include *ERBB2* amplification with *TP53* mutation, and to a lesser extent with *CDKN2A* aberrations [41]. Events that co‐occur less often than expected are *BRAF* mutations with *ERBB2* amplification, *TP53* mutation, or *KRAS* mutation. MOC are very rarely haploid, supporting a potential germ‐cell origin only for a few exceptional cases (in addition to the rare association with teratomas) [84].

**Table 1:** Multigene mutation and copy number studies of MOC and MBT

Study	Platform	Number MOC	Number MBT	Comment	Genes >10% in MOC
Dundr [60]	TSP (727 genes) + fusions	29 + 14 equivocal for MOC	49		*KRAS, TP53, CDKN2A, ERBB2* (amp)
Simons [73]	TSP (56 genes)	7	13 + 10 benign	13 teratoma associated, 17 Brenner tumour associated	*KRAS, TP53, ELF3, CDKN2A* (HD/Mut)
Cheasley [5]	WES + WGS + TSP (508 genes)	48 WES, 5 WGS, 134 TSP	9 WES, 20 TSP	Comparison to STAD/COAD/UCEC (diff) & PDAC (similar)	*KRAS, TP53, RNF43, CDKN2A, ERBB2* (amp)
Mueller [74]	WES + TSP (351 genes)	9	15	Comparison to STAD/COAD/UCEC (diff) & PDAC (similar)	*KRAS, TP53, CDKN2A, ARID1A, PIK3CA, ERBB2* (amp)
Meagher [75]	TSP (45 genes)	128	28	Comparison to other mucinous tumours	*KRAS, TP53, PIK3CA*
Ryland [76]	WES	11	8	Overlap with Cheasley et al 2019	*KRAS, TP53, RNF43, CDKN2A, BRAF*
MacKenzie [77]	TSP (Hotspot 50 genes) and IHC	37	26		*KRAS, TP53, CDKN2A, PIK3CA, ERBB2* (amp)
Chay [78]	TSP (MassArray hotspot 19 genes)	45	0	Lower % with *KRAS* in Asian population	*KRAS* (45%)

Abbreviations: amp, amplified; COAD, colorectal adenocarcinoma; diff, different; HD/mut, homozygous deletion/mutation; IHC, immunohistochemistry; PDAC, pancreatic adenocarcinoma; STAD, stomach adenocarcinoma; TSP, targeting sequencing panel; UCEC, uterine carcinoma; WES, whole‐exome sequencing; WGS, whole‐genome sequencing.

A comparison of the MOC mutation profile with other mucinous tumours found clear differences compared to mucinous colorectal and appendiceal tumours, with MOC lacking the *APC* mutations that are common in these tumour types [5]. There are some molecular similarities with pancreatic ductal adenocarcinoma (PDAC); both tumour types share somatic genetic events in *KRAS*, *TP53*, and *CDKN2A*. However, MOC lacked *SMAD4* mutations, which are common in PDAC, while *ERBB2* amplifications are rare in PDAC. In addition, *ERBB2* amplifications were more common in the expansile growth pattern of MOCs compared to the infiltrative growth pattern [56, 60], correlating with earlier studies that found *ERBB2* amplifications are associated with a better clinical outcome [85]. From a histological perspective, and given the frequency of *ERBB2* amplification, MOC are similar to adenocarcinomas of the gastroesophageal junction [86]. These tumours arise through metaplastic change (Barrett's oesophagus) with the appearance of goblet cells perhaps similar to MBT/MOC.

The mutational signatures of MOC do not point to any particular exposure or mechanism that could link it to a cell of origin or causal mutational process, with most mutations attributed to age‐related signatures [5]. Despite the epidemiological link with smoking, no dominant smoking signatures have been detected. *KRAS* hot‐spot mutations vary between cancer types: for MOC, the most common variants are G12D (39%) and G12V (33%), distinct from lung adenocarcinoma (in which G12C is the most common at 41%), and similar but not identical to colorectal, pancreatic, and stomach adenocarcinomas (Figure 2B). Colorectal and stomach adenocarcinomas have more G13D mutations compared to MOC, while pancreatic adenocarcinomas have more G12R. The proportion of transition versus transversion variants in MOC was similar to the gastrointestinal tumours, but distinct from lung cancers (more transversions in lung cancer). These patterns suggests that the mutational exposures are similar but not identical to these gastrointestinal cancers. Interestingly, the mutation profile for a combination of low‐grade serous ovarian carcinomas and serous borderline ovarian tumours is near‐identical to MOC, suggesting a related origin (Figure 2B).

### Gene expression

MOC have consistently been shown to be outliers in gene expression studies of EOC [87, 88], although most early studies only evaluated a handful of cases, limiting the capacity to identify robust signatures of differentially expressed genes between MOC and other EOC histotypes (Table 2). In addition, no gene expression studies have so far identified MOC molecular subtypes. Nonetheless, studies have identified a few genes that are commonly upregulated in MOC (e.g. *LGALS4*, *CAECAM5*, and *TFF1*). More recently, a large study using a targeted NanoString‐based panel identified genes that could distinguish MOC/MBT from lower‐gastrointestinal tumours (GI, colorectal, and appendiceal) [56]. In contrast, upper‐GI tumours (pancreatic, gastric) were not able to be distinguished with any accuracy. However, the panel identified strongly prognostic genes, including *TAGLN* and *THBS2*, which were associated with shorter overall survival and the infiltrative growth pattern.

**Table 2:** Gene expression studies of MOC

Study	Number MOC	Other EOC	Other tissues	Findings
Meagher [56]	333 (+151 MBT)	‐	COAD, PDAC, APP	19 gene panel could not distinguish MOC from upper‐GI tumours. *THBS2* and *TAGLN* prognostic
Elias [89]	9	13 HGS	PGC, PDAC, MCN, FT, OSE	Similar gene expression between MCN, MOC and PGC
	(+8 MBT)			
Engqvist [90]	10	86 HGS, En, CC		MOC clustered together, were most similar to En
Marquez [87]	9	SOC, En, CC	FT, OSE, Col	MOC most differentially expressed genes; more similar to Col than FT/OSE.
Heinzelmann‐Schwartz [91]	3	SOC, En, CC, SBT	Whole ovary	MOC distinct from other histotypes
	(+4 MBT)			
Jochumsen [92]	1	SOC, En, CC		Intratumoural heterogeneity slightly higher in MOC than others
Wu [93]	9	HGS, En, CC		Not analysed separately
Ramakrishna [94]	6	SOC, En, CC		Not analysed separately
Mateescu [95]	8	SOC, En, CC		Not analysed separately
Huang [96]	14 (+5 MBT)	SOC, En, CC		Copy number aberrations distinct to MOC correlated to gene expression (e.g. *ERBB2*)
Wang [97]	14 + 27 validation	LGS, En, CC		Survival signature across rare histotypes but not in public HGS data
Wu [98]	13	SOC, En, CC		Sample overlap with Schwartz [91]; MOC distinct from other histotypes

Abbreviations: APP, appendiceal adenocarcinoma; CC, clear cell; COAD, colorectal adenocarcinoma; Col, colon mucosa; En, endometrioid; EOC, epithelial ovarian carcinoma; FT, fallopian tube; HGS, high‐grade serous; LGS, low‐grade serous; MBT, mucinous borderline tumour; MCN, mucinous cystic neoplasms; MOC, mucinous ovarian carcinoma; OSE, ovarian surface ‘epithelium’; PDAC, pancreatic ductal adenocarcinoma; PGC, primordial germ cells; SBT, serous borderline tumour; SOC, serous (unspecified).

A combined pan‐cancer analysis has not been performed so far to search for gene expression similarities between MOC and other cancer types. However, Marquez *et al* [87] included normal colonic mucosa in their analysis, which was more similar to MOC than MOC was to ovarian surface epithelium or fallopian tube epithelium. This similarity in morphology and gene expression, while suggesting some functional analogy, has not helped with identifying a cell of origin. One recent study proposed a novel origin hypothesis for MOC based on gene expression signatures [89], suggesting that MOC and mucinous cystic neoplasms (MCN) of the pancreas are derived from primordial germ cells. During embryogenesis, these cells migrate through the embryo and pass close by the developing pancreas before settling in the ovary. MOC and MCN show some interesting similarities: MCN occurs almost exclusively in women and is the only extra‐ovarian tumour in the body that contains ovarian‐type stroma. It is also hypothesised that the ovarian‐type stroma, perhaps induced by the misplaced germ cells, might play a role in inducing mucinous epithelium formation [99]. The two tumours also share genetic aberrations such as *KRAS* and *RNF43* mutations. Such an intriguing hypothesis, like many regarding cell of origin, is difficult to demonstrate definitively, but could be strengthened by techniques proposed to elucidate lineage more strongly than gene expression, such as those assessing chromatin structure (e.g. ATACseq). In addition, transgenic animal models could also be harnessed to see whether oncogenes driven by key cell type‐specific transcription factors could drive tumour formation.

### Epigenetic profiling

Epigenetic studies of MOC are few (Table 3), despite the insights such an analysis might confer about the cell of origin, given epigenetic marks and chromatin structure could be more stable and representative of the cell of origin than RNA [104]. Most studies to date have included MOC in pan‐ovarian cancer analyses. Not surprisingly, these studies have found MOC to be distinct. Some studies showed a greater similarity to either endometrioid or clear‐cell ovarian carcinoma, but with less extensive hypermethylation. However, no studies have been well‐powered to compare MOC to potential cells of origin.

**Table 3:** Genome wide methylation studies

Study	Number MOC	Other	Method	Conclusions
Cicek [100]	12	473 other EOC	Infinium 450 Bead array	Focused on a CC predictor
Yoon [101]	3	14 other EOC, 5 normal ovary	Illumina Human‐6 v2 Expression BeadChip	MOC had a distinct methylation profile to other EOC
Lieuw [102]	24	103 SOC, 337 non‐EOC (COAD, STAD, non‐GI), 6 benign MOT	MethylCap‐seq and Infinium 450 Bead array (some from Yamaguchi 2014 GSE51820)	Identified 101 differentially methylated genes compared to SOC. Proteosome identified as reduced methylation in MOC
Bodelon [103]	16	76 SOC, 33 EnOC, 7 CCOC, 30 SBT	Infinium 450 Bead array	MOC clustered in mixed group with SOC and EnOC
Engqvist [90]	11	51 SOC, 17 EnOC, 17 CCOC	Infinium Methylation EPIC BeadChips	MOC clustered with CCOC and EnOC but is less hypermethylated

Abbreviations: C, colon mucosa; CCOC, clear‐cell ovarian carcinoma; COAD, colorectal adenocarcinoma; EnOC, endometrioid ovarian carcinoma; EOC, epithelial ovarian carcinoma; FT, fallopian tube; GI, gastrointestinal; MOC, mucinous ovarian carcinoma; MOT, mucinous ovarian tumour; O, whole ovary; OSE, ovarian surface ‘epithelium’; SBT, serous borderline tumour; SOC, serous ovarian carcinoma; STAD, stomach adenocarcinoma.

## Clonal analysis of rare precursors

In attempting to identify a cell of origin for MOC, several studies have looked for genetic relationships within a single patient (clonality) for suggested precursors, such as teratomas and Brenner tumours (Table 4). The challenge with these studies is the generally limited resolution of the platforms used and the quiet genomic profiles of benign Brenner tumours and teratomas. The combination of these factors reduces the power of such analyses. Comprehensive whole‐genome sequencing of tumours with matching normal DNA would be required for a more accurate estimation.

**Table 4:** Studies exploring clonality with coexisting lesions

Study	Platform	Number MOC	Number MBT / benign	Lesions compared to	Findings	Genes shared
Simons [73]	TSP (56 genes); CN by LC‐WGS	13 by TSP, 8 CN only	7 MBT, 10 benign (TSP); 21 MBT, 17 benign (CN)	24 Brenner (13 by TSP)	2 Brenner tumours clonal with (a) benign mucinous and (b) MBT.	*CDKN2A*
				22 Teratomas (17 by TSP)	No teratomas clonal	
Tafe [105]	TSP (358 genes)	0	2 MBT, 4 benign	Brenner	Brenner: 2 clonal by CN; no shared driver mutations—other shared variants likely germline	NA
Wang [106]	X inactivation/ STR	5	3	Brenner	8/8 concordant	NA
Mesbah Ardakani [107]	TSP (26 genes)	7	0	Mural nodules	All clonal	6 *KRAS*, 1 *TP53*
Zhang [108]	*KRAS* only	0	1	Benign mesonephric‐like lesion	Clonal, but different IHC profiles	*KRAS*
Nilforoushan [109]	TSP (149 genes)	0	1	Mesonephric neoplasm	Clonal, mesonephric neoplasm had more variants	KRAS
Da Silva [110]	TSP (468 genes)	0	2	Mesonephric‐like carcinoma	Clonal	*KRAS*, CN
Choi [111]	WES (PMP) *KRAS*, *GNAS* (Teratoma)	1	0	Teratoma, PMP	Teratoma + PMP nonclonal; solid mucinous tumour not analysed	NA
Snir [112]	STR	6	0	Teratoma	5/6 concordant	NA
Hershkovitz [113]	*KRAS*	1	0	Teratoma	nonconcordant	NA
Li [114]	*KRAS*	2	0	Teratoma	nonconcordant	NA
Wang [115]	X inactivation/ STR	2	3 MBT, 1 benign	Teratoma	5/6 concordant	NA
Kerr [116]	STR	5	0	Teratoma	4/5 concordant	NA
Fujii [117]	STR	3	0	Teratoma	2/3 concordant	NA
Kato [118]	MS‐MLPA, KRAS, ERBB2	14 (3 with teratoma)	14 (4 with teratoma	Teratoma	Similar methylation profiles in 9 imprinted genes; *KRAS*/*ERBB2* not shared	9 genes

Abbreviations: CN, copy number; LC‐WGS, low coverage whole‐genome sequencing; MS‐MLPA, methylation specific multiplex ligation‐dependent probe amplification; PMP, pseudomyxoma peritonei; STR, short tandem repeat; TSP, targeted sequencing panel; WES, whole‐exome sequencing.

### Teratomas

The development of mucinous ovarian tumours in the context of an associated teratoma is uncommon (3%–8% of mucinous tumours) [119]. Intriguingly, teratomas observed with mucinous tumours can be associated with pseudomyxoma peritonei, even in the absence of any appendiceal lesions [119, 120]. Furthermore, teratoma‐associated ovarian mucinous tumours often show a lower GI/appendiceal IHC profile (CK7−, SATB2+) [119]. Accordingly, this led to a proposal to use a separate terminology for teratoma‐associated ovarian mucinous neoplasm—low‐grade ovarian mucinous neoplasm arising in teratoma (LOMN)—akin to appendiceal mucinous neoplasms (i.e. low‐grade appendiceal mucinous neoplasm [LAMN]) [3]. Hence, in the future, teratoma‐associated ovarian mucinous neoplasms will be categorised separately.

Low‐resolution single tandem repeat (STR) and X‐inactivation studies indicated a clonal relationship between coexisting teratoma and mucinous tumour components in 14/18 cases [112, 115, 116, 117]. Notably, 18/23 (78%) of these clonal cases demonstrated pseudomyxoma and/or SATB2+/CK7− disease, consistent with LOMN. However, in separate studies none of three independent cases shared *KRAS* mutations between the teratoma and mucinous tumour epithelia [111, 113, 114], and a targeted sequencing panel combined with copy number analysis found no clonal cases in the 22 studied [73]. The development of a mucinous ovarian tumour from a teratoma may require acquisition of mutations in *KRAS* and/or *GNAS* (common in LAMN), not observed in the teratoma itself [73, 111].

### Brenner tumours

The association of mucinous tumours with benign Brenner tumours is well documented. A careful evaluation of 40 mucinous benign cystadenomas showed that 25% contained a Brenner component [121]. Borderline mucinous tumours are also occasionally observed [73]. Of note, this is rarely evident in MOC, possibly due to overgrowth of this component or lack of progression potential (‘evolutionary cul‐de‐sac’). Brenner tumours are thought to arise from transitional/urothelial‐type epithelium, which commonly occurs in the ovary as so‐called Walthard nests. Epidemiologically, compared to mucinous tumours, Brenner tumours are seen in older women (mean age 61) [73], but more information on other risk factors is lacking. Urothelial carcinomas (which have the same epithelium type as Brenner tumours) and mucinous tumours share smoking as a risk factor.

Clonal relationships by X‐inactivation markers were observed in 8/8 Brenner tumours coexisting with MOC or MBT [106]. Two studies performed more extensive clonality analysis using panel sequencing and together found that 3/30 Brenner tumours shared a copy number event and 1/19 a somatic mutation with the coexisting mucinous tumour [73, 105]. Despite this evidence for clonal relationships between coexisting mucinous and Brenner tumours, there were no consistent genetic events across all mucinous tumour types that would indicate a driver of mucinous metaplasia from a Brenner precursor, but perhaps this alteration could be driven by heterogeneous alterations or due to a microenvironmental stimulus. Since mucinous metaplasia can occur in benign Brenner tumours independent of a coexisting mucinous tumour, it is plausible that mucinous metaplasia in Brenner tumours is independent of any mutations, but that *KRAS* mutation (and/or another event in mucinous tumour pathogenesis, such as in *CDKN2A*) causes expansion of the mucinous epithelium to form a mucinous neoplasm.

## Potential origins for MOC not associated with teratoma or Brenner tumours

**Figure 3:**
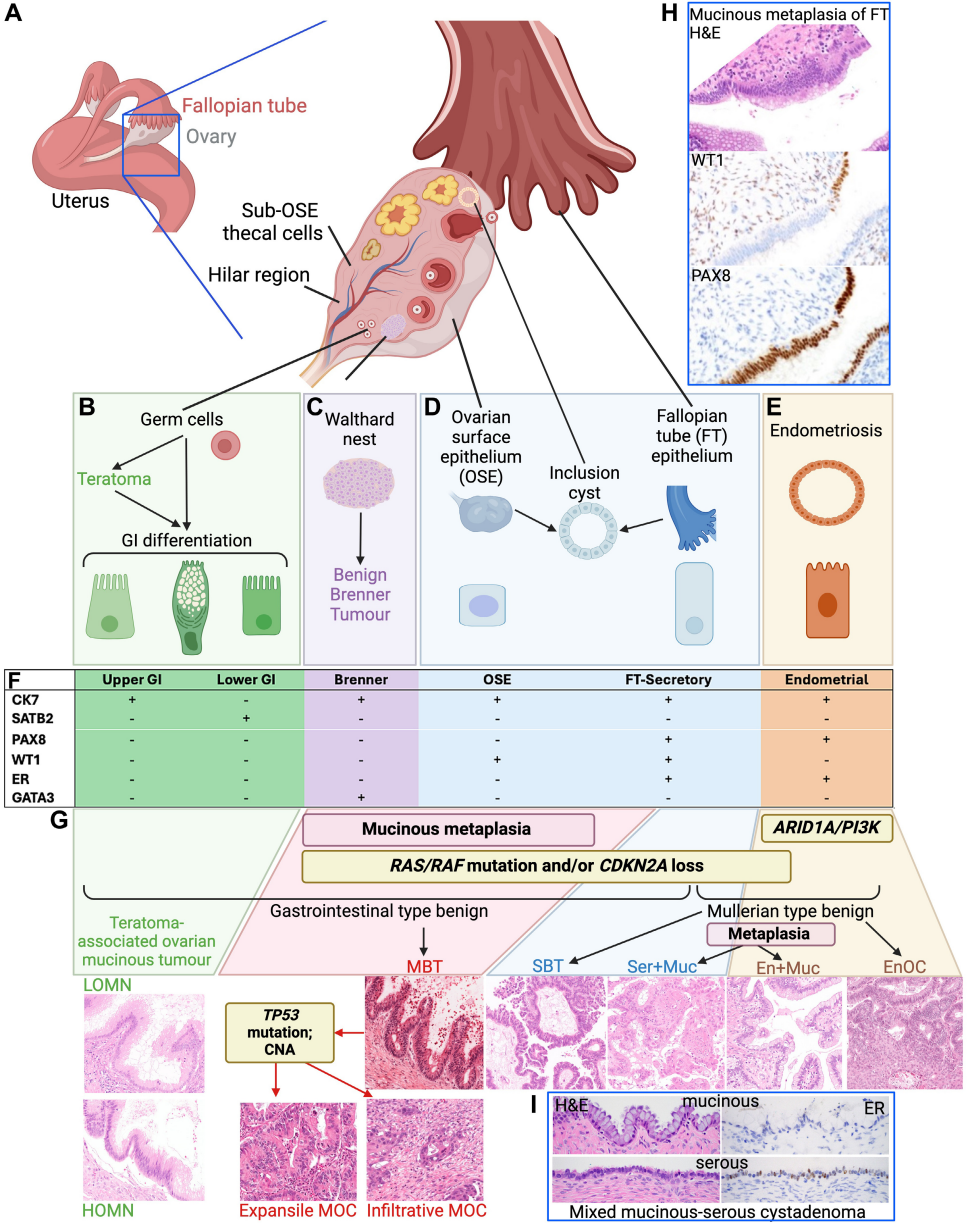
Potential cells of origin for MOC. (A) Anatomic depiction of the gynaecologic tract, showing relevant cell types. (B) A subset of mucinous tumours at the ovary may develop from germ cells, with evidence for GI differentiation to arise via a teratoma intermediate. These become teratoma‐associated low‐grade or high‐grade ovarian mucinous neoplasms (LOMN and HOMN respectively). (C) Similarly, some mucinous tumours may arise from Brenner tumours, from cells of origin in Walthard nests (whose origin is itself unknown). (D) Entrapped fallopian tube (FT) epithelium (or possibly ovarian surface ‘epithelium’ (OSE), sub‐OSE stem cells or hilar stem cells form an inclusion cyst within the ovary, which may undergo metaplasia to include mucinous‐type cells (see H below). (E) Endometriosis can form in the ovary or elsewhere, and comprises endometrial‐type epithelium. (F) Key immunohistochemical markers related to each cell type from B–E. (G) Progression models for mucinous and mucinous‐associated tumours of the ovary. LOMN and HOMN form from teratomas; mucinous metaplasia occurring in Brenner tumours, FTE, inclusion cysts or benign cystadenomas leads to a benign gastrointestinal type epithelium that further evolves into mucinous borderline tumours (MBT) and MOC, driven by genetic events in *KRAS* and/or *CDKN2A*. Mutations in these genes in Müllerian‐type benign epithelium (e.g. inclusion cyst or serous cystadenoma with no mucinous metaplasia) leads to serous borderline tumours (SBT) and low‐grade serous carcinomas. The inclusion cyst pathway could therefore be a shared origin with serous tumours and could also result in rare mixed serous/mucinous tumours (Ser + Muc). Mucinous metaplasia could also occur in a benign tumour arising from endometriosis, leading to the ‘seromucinous’ type, more correctly an endometrioid tumour with mucinous differentiation (En + Muc). These tumours may have *KRAS* mutations but are also likely to carry mutations in *ARID1A* and/or *PIK3CA*. Of course, endometriosis with mutations in these latter two genes also leads to endometrioid ovarian carcinomas (EnOC). (H) Example of mucinous metaplasia of the FT showing the mucinous component lacks WT1 staining but maintains PAX8. (I) Example of benign cystadenoma, comprising both serous and mucinous‐type epithelia, the mucinous component lacks ER staining. Created with BioRender.com

A number of other hypotheses on the origins of MOC have been proposed (Table 5). While the putative cells of origin of the other main ovarian carcinoma histotypes are readily observed in noncancerous tissues, such as (a) endometriosis (ectopic endometrium) for endometrioid or clear‐cell carcinoma; (b) endosalpingiosis (ectopic fallopian tube‐type epithelium in ovarian inclusion cysts) for low‐grade serous neoplasms and serous borderline tumours; and (c) eutopic fallopian tube epithelium in the fallopian tube fimbria for high‐grade serous carcinomas; normal gastrointestinal‐like epithelium in non‐neoplastic ovaries is not obvious. However, a careful evaluation of 403 completely embedded grossly normal ovaries revealed benign mucinous epithelium within ‘surface epithelial inclusions’ in 5% [135]. While it is not entirely clear from which cell type this mucinous epithelium has arisen, the presence of cysts lined by both tubal type epithelium (very common in inclusion cysts) and mucinous epithelium (rare) suggests the origin of the latter from the former via metaplasia in response to unknown stimuli [122] (Figure 3). Similarly, mucinous metaplasia is rare but well documented with the fallopian tube [136] and other gynaecological tissues in the context of Peutz–Jeghers syndrome (*STK11* germline mutation) [134]. This latter association shows the possibility of mucinous metaplasia from other types of Müllerian epithelia within the ovary, although *STK11* mutations are very rare in MOC. Such gastrointestinal‐type mucinous metaplasia must be distinguished from unrelated seromucinous neoplasms, which are of endometrioid cell lineage (WT1−, PR+), but show an admixture of ciliated and Müllerian (i.e. endocervical‐type, hormone receptor‐positive) mucinous cells. It is very unlikely that gastrointestinal‐type mucinous neoplasms arise from endometriosis, given the lack of association and epidemiological data.

**Table 5:** Potential cells and lesions of origin of mucinous ovarian tumours

Cell type/location	Epidemiology	Histopathology	Genetics	IHC/expression markers of the cell type	Comments
Inclusion cysts	Very rare before menarche, increase with age	~5% of non‐neoplastic ovaries have mucinous metaplasia in inclusion cysts [122]		PAX8+, ER+, WT1+	Lacking data but plausible origin for both MOC and LGSC.
Fallopian tube epithelium	Endosalpingiosis common (7.6% in women undergoing laparoscopy), and associated with increased risk of gynae malignancy. [123]	Mucinous metaplasia in 3% FTs [124, 125]; primary FT MOC has been observed [126]		PAX8+, WT1+ secretory cells	MOC rarely PAX8, but expression could be lost during neoplastic growth
Ovarian surface cells				CALB2+, WT1+	Amhr2‐Cre transgenic mouse model supports OSE origin
Brenner tumours/transitional cells/Walthard nests	Brenner tumours tend to be in older women	Co‐associated, but not all MOC have Brenner	Clonality observed with Brenner tumours—*CDKN2A* (Table 4)	CK7+, GATA3+, P63+, PAX8‐, CK20‐, ER‐	Good evidence for clonality
Teratomas		Co‐associated, but few MOC have teratoma	Rare clonal events with low‐resolution methods (Table 4); uncommon haploidy in MOC [84]	Sometimes contain GI‐type epithelium that expresses CK20 and SATB2 = appendiceal‐like IHC profile [73]	Unlikely for majority
Ovarian hilar/ junctional region or other ovarian region		Mucinous tumours arise in abnormal ovaries—hypocellular cortex and reduced primordial follicles [127]		Express stem cell markers [128]	Lacking evidence
Primordial germ cells	Connection to pancreatic mucinous cystic neoplasms (female bias)		Genetics of MCN somewhat different	Shared gene expression	Correlative evidence only
Wolffian ducts/ mesonephric remnants	Coexistence of mesonephric‐like lesions (ML) in post‐menopausal patients	Coassociated, but not all MOC have ML	Clonal mesonephric lesions with MBT; frequent *KRAS* mutations and 1q gains (Table 4)	GATA3+, PAX8+, CK7+	Good evidence for clonality, although IHC profile is different. Alternatively, could arise from common Müllerian precursor. ML also seen with LGSC
Endometrial mucinous metaplasia	No decrease in MOC with tubal ligation [129]		Associated with *KRAS* mutations [130]	ER+	MOC uncommonly ER+. Maybe for ‘seromucinous’ type but not likely for the rest
Cervical metaplasia/ gastric‐type adenocarcinoma	No decrease with tubal ligation [129]. Associated with Peutz–Jeghers syndrome [131]	Cervical gastric‐type adenocarcinoma looks similar [131]	*STK11* mutations are rare in MOC; *KRAS* mutations rare in cervical [132]		Unlikely—different mutation profiles
Developmental müllerianosis		Developmental adenomyosis, endometriosis, and endocervicosis observed in fetuses [133]			Speculative
Synchronous mucinous metaplasia and neoplasia of the female genital tract		Ovarian mucinous tumours observed in this syndrome [134]	*STK11* mutations associated		*STK11* mutations are rare in MOC

Abbreviations: FT, fallopian tube, GI, gastrointestinal; MBT, mucinous borderline tumours; MCN, mucinous cystic neoplasms (of the pancreas); ML, mesonephric‐like lesions; MOC, mucinous ovarian carcinoma.

Oncogenic *KRAS* mutations are very common in borderline tumours, both mucinous and serous, and it is plausible that most cases of both neoplasms arise from ovarian inclusion cysts. It is unclear how these two histologically distinct tumours arise from the same cell of origin, although this scenario is analogous to the development of clinicopathologically distinct neoplasms with similar genetics (endometrioid and clear‐cell carcinomas) from another common precursor lesion (endometriosis). The metaplastic switch from serous tubal type epithelium to mucinous epithelium could occur prior to *KRAS* mutation, but with this event leading to expansion of either the serous or mucinous cyst with subsequent development of a borderline tumour of the same type.

Alternatively, a *KRAS* mutation in the appropriate cellular and microenvironmental context may cause a reprogramming of a differentiated secretory or ciliated cell back into a stem cell, allowing for transformation along a mucinous cell lineage, which has been shown for alveolar lung epithelium [137]. This idea is further supported by the observation of a case of a so‐called ‘ovarian RASoma’, which was composed of mesonephric, mucinous, and endometrioid components, all clonally linked by the same G12A *KRAS* mutation [138]. A similar dilemma of a missing link to a cell of origin has evolved for the recently described mesonephric‐like adenocarcinomas of the endometrium and ovary [139]. These tumours cluster by methylation profiling together with mesonephric‐type adenocarcinomas of the uterine cervix, which arise from mesonephric remnants [140]. However, the endometrial and ovarian counterparts do not seem to arise from mesonephric remnants. Instead, they are associated with a variety of Müllerian lesions, including endometriosis/endometrioid neoplasms, low‐grade serous and mucinous neoplasms (all MAPK mutation‐related). The current hypothesis is that these mesonephric‐like adenocarcinomas arise from other Müllerian neoplasms through transdifferentiation. Overall, there is accumulating evidence of the possibility of (multi)directional metaplasia of benign Müllerian tissue or transdifferentiation of a Müllerian neoplasm along various non‐Müllerian lineages (mesonephric, gastrointestinal, mucinous), especially in the context of *KRAS* mutations. Indeed, four mesonephric neoplasms with mucinous borderline tumour components were clonally related, sharing *KRAS* mutations (Table 4) [108, 109, 110].

One of the more plausible theories is neoplastic initiation in inclusion cysts. This idea is consistent with the intra‐ovarian presentation of mucinous tumours and the observation of mucinous metaplasia in these lesions. The observation of both serous and mucinous differentiation within these lesions also leads to the question of whether inclusion cysts are a common ancestor for serous and mucinous borderline tumours (and the carcinomas that develop from these). There are parallels with their genetic events (*KRAS*, *CDKN2A*) and location. Indeed, the sole transgenic mouse model that develops mucinous tumours (AMH2R‐Cre driven *KRAS* and *PTEN* mutations on a *TP53* heterozygous mutant background) also develops serous tumours [141]. Perhaps after enclosure within the ovarian stroma, exposure to hormones and/or mutational events leads the cells within these cysts to develop neoplasia down one or other of the histologies. Benign serous cystadenomas frequently contain an adenofibroma component (i.e. cystadenofibromas) that carry independent copy number events, and it has been suggested that the early initiation of these tumours involves crosstalk between these nonclonal components [142]. This relationship has not been described for mucinous tumours, but perhaps an abnormal ovarian structure, such as that seen associated with MBTs [127], provides a different microenvironment that favours mucinous differentiation.

Despite the observations above, the origin of the epithelial cells within these cysts is unclear: are these derived from fallopian tube epithelial cells [143] or mesothelial cells from the surface of the ovary (commonly referred to as ‘ovarian surface epithelium’ despite being a different cell type) [144]? For these origins, the theory is that during ovarian rupture while ovulating, fallopian tube or ovarian surface epithelia are ‘captured’ by the ovarian stroma. This idea is supported histologically by ovarian epithelial clefts with these cell types, but as an origin for mucinous tumours the weak epidemiological association with factors associated with the number of ovulatory cycles reduces this likelihood [28]. Alternatively, do cyst cells come from another cell type, such as transitional cells, mesonephric remnants, hilar stem cells, or primordial germ cells? Gene expression, immunohistochemistry, and histological studies have been inadequate to answer this question, providing only correlative evidence. A mouse model evaluating cellular lineage using *OVGP1*‐driven fluorescent tagging found inclusion cysts and endosalpingiosis expressing the fluorescent tag and interpreted this as nonfallopian tube origin based on the short lag time between induction of fluorescence and the observation of these lesions, as well as the lack of association with age and ovulation [145]. Whether mouse biology and anatomy are sufficiently similar to humans to make such a conclusion is not certain. It is also unclear why mucinous differentiation arises in inclusion cysts at all—what are the drivers for this cellular phenotype? Mucins are generally produced when required for lubrication—but why would this be stimulated by an ovarian stroma?

## Summary and future directions

Despite all that has been learned in recent years, the MOC field remains plagued with uncertainty: substantial gaps persist regarding its origins, diagnosis, and treatment. The central hindrance is the rarity of the disease. This challenge requires concerted, collaborative efforts. Establishment of an international registry would be beneficial to drive future research work forward, including for cell of origin research by increasing the number of cases included in epidemiological and genetic studies. Such a registry requires collaborations between research institutions and will need to overcome barriers to data sharing and analysis in compliance with local regulations. Federated learning is a novel artificial intelligence (AI)‐based collaborative approach that can be leveraged to overcome data‐sharing barriers by learning without sharing patient data [146]. The research should prioritise the development of secure and interoperable platforms for data aggregation and analysis. By overcoming the data‐sharing hurdles, we will ensure better global representation.

An international registry could also support planning of future prospective studies by identifying populations of interest for trial recruitment, translational research, and drug discovery. This resource would improve the feasibility of primary evidence generation through registry and pragmatic trials. Comprehensive evaluation of treatment efficacy is paramount in addressing the observed variability in treatment responses among MOC patients, particularly given previous trends in treating MOC with lower gastrointestinal regimens, which has little supportive evidence [8]. Elucidating optimal treatment strategies tailored to an MOC invasion pattern and/or molecular subtypes and patient characteristics will improve patient outcomes and quality of life. Understanding the cell of origin for the various mucinous ovarian tumours could be central in devising novel treatment strategies, such as those targeting key transcription factor programs [147].

Advancing MOC research also necessitates differentiating between primary tumours and upper‐GI metastases to ensure that cohorts contain ‘true’ primary MOC. AI could help to distinguish MOC from GI metastases based on patterns learned from digital pathology images [148]. This approach could complement the discovery of novel diagnostic omics‐based biomarkers, particularly preoperative circulating or imaging‐based biomarkers.

We have presented here models of origin and progression for different types of mucinous tumours at the ovary; however, these remain mostly unproven. Large cohorts of carefully curated and well annotated tumours, normal and precursor tissues, analysed using integrated multi‐omic, single cell, and spatial methods will be needed to definitively address the cell of origin question. Evidence from sophisticated cellular and animal models will help to validate associations identified through such studies.

## Author contributions statement

This study was conceived at Ovarian Tumor Tissue Analysis (OTTA) Consortium meetings in 2019 and 2024, through discussions involving KLG, NSM, SJR, MSA, ANK, AB, AT, PMW and MK. All authors contributed to drafting and revising the article. Figures were prepared by NSM, KLG, ANK, MK and PPDP.

## Supporting information


**Table S1.** SNP odds ratios for mucinous ovarian carcinoma and mucinous borderline tumours

## Data Availability

Data sharing is not applicable to this article, as no new data were created or analysed in this study.
