# Supplementary material for: Cellular origins of mucinous ovarian carcinoma
Source: J Pathol. 2025 Mar 3;266(1):9–25. doi: 10.1002/path.6407 (PMC11985703; doi:10.1002/path.6407)
Supplement: Supplementary file 1 — Table S1. SNP odds ratios for mucinous ovarian carcinoma and mucinous borderline tumours [file PATH-266-9-s001.docx]

**Cellular origins of mucinous ovarian carcinoma**

NS Meagher *et al. J Pathol* <https://doi.org/10.1002/path.6407>

**Table S1.** Odds ratios (OR) and 95% confidence interval (CI) for single nucleotide polymorphisms (SNPs) in mucinous ovarian carcinoma (MOC, *n =* 1,417) and mucinous borderline tumours (MBT, *n =* 1,149) separately for those loci for which this analysis was done; OR is shown in the positive direction for the risk SNP regardless of allele frequency (Pharoah, Berchuck, unpublished).

| Locus | SNP | Risk allele frequency | MOC OR (95%CI) | MBT OR (95%CI) |
| --- | --- | --- | --- | --- |
| 2q13 | rs752590 | 0.21 | 1.27 (1.16–1.39) | 1.33 (1.20–1.48) |
| 2q31 | rs711830 | 0.32 | 1.26 (1.17–1.37) | 1.28 (1.17–1.40) |
| 3q23 | rs112071820 | 0.28 | 1.27 (1.17–1.39) | 1.31 (1.19–1.45) |
| 9q31.1 | rs320203 | 0.85 | 1.30 (1.15–1.46) | 1.27 (1.11–1.45) |
| 19q13.2 | rs688187 | 0.31 | 1.33 (1.21–1.46) | 1.58 (1.41–1.76) |
